# Myristic acid beneficially modulates intervertebral disc degeneration by preventing endplate osteochondral remodeling and vertebral osteoporosis in naturally aged mice

**DOI:** 10.3389/fphar.2025.1517221

**Published:** 2025-04-01

**Authors:** Yan Gong, Yuzhuo Zhang, Xingda Chen, Zelin Zhou, Weicheng Qin, Yanchi Gan, Jiahui He, Jizhi Ma, Guifeng Chen, Qi Shang, Kai Tang, Honglin Chen, Yu Liu, De Liang, Gengyang Shen, Xiaobing Jiang, Zhaojun Cheng

**Affiliations:** ^1^ Trauma and Orthopaedics Center, The First Affiliated Hospital of Guangzhou University of Chinese Medicine, Guangzhou, China; ^2^ Department of Traditional Chinese Medicine, The Second Affiliated Hospital of Guangzhou Medical University, Guangzhou, China; ^3^ Department of Orthopedics, Ruikang Hospital Affiliated with Guangxi University of Chinese Medicine, Nanning, China; ^4^ Department of Orthopedics, The Affiliated TCM Hospital of Guangzhou Medical University, Guangzhou, China; ^5^ Department of Orthopedics, Shanghai Key Laboratory of Orthopedics Implant, The Ninth People’s Hospital, Shanghai Jiao Tong University School of Medicine, Shanghai, China; ^6^ Department of Spinal Surgery, The Second Affiliated Hospital of Guangzhou Medical University, Guangzhou, China; ^7^ Lingnan Medical Research Center, Guangdong Clinical Research Academy of Chinese Medicine, Guangzhou, China; ^8^ Postdoctoral Research Station, Guangzhou University of Chinese Medicine, Guangzhou, China

**Keywords:** myristic acid, endplate chondrocytes, osteoporosis, endplate osteochondral remodeling, intervertebral disc degeneration

## Abstract

**Background:**

The origin of intervertebral disc degeneration (IDD) is highly complex, where both cartilage endplate remodeling and vertebral osteoporosis are of utmost importance. Myristic acid (MA), a saturated fatty acid derived from nutmeg, a traditional Chinese herb, has been shown to boost memory. Additionally, its isomers have been verified to have anti-osteoporotic characteristics. However, the precise mechanism by which MA functions in relation to IDD remains unclear.

**Methods:**

*In vivo*, a naturally aged animal model was used. The drug—administration method of MA was intraperitoneal injection to mice aged 22 months at a dose of 2 mg/kg·d for 2 months. Micro-CT observed vertebral bone mass and endplate changes, followed by Hematoxylin‒eosin (H&E), Masson, and Safranin-O staining of tissues. TRAP staining counted osteoclasts; immunohistochemistry detected the expressions of Aggrecan and Collagen II. Bioinformatics explored MA’s anti-IDD mechanism. *In vitro*, MA-treated senescent endplate chondrocytes (induced by TBHP) were analyzed by RT-qPCR and immunofluorescence (IF) for senescence and matrix synthesis markers. TRAP and F-actin detected MA’s effect on RAW264.7 osteoclast differentiation (induced by RANKL); qPCR examined the expressions of osteoclast genes.

**Results:**

Using the naturally aged model, we found that MA tended to improve vertebral osteoporosis and endplate osteochondral remodeling, decreased the TRAP activity of the endplate, and alleviated IDD in naturally aged mice. Bioinformatics analysis suggested that the relationships among IDD, osteoporosis, and endplate degeneration were mainly linked to cellular senescence. *In vitro*, MA postponed the senescence of TBHP-induced endplate chondrocytes by increasing the expression of Aggrecan and decreasing the expressions of MMP-3, MMP-9, and the senescence markers p16 and p21. Additionally, MA notably inhibited osteoclast activity, as evidenced by a decrease in the number of osteoclasts and a significant suppression of F-actin formation. At the molecular level, MA efficiently reduced the expressions of osteoclast marker genes like ACP-5, CTSK, and DC-STAMP.

**Conclusion:**

The findings of this research suggest that MA is capable of inhibiting endplate osteochondral remodeling and vertebral osteoporosis, diminishing osteoclastogenesis to preserve bone mass, and consequently delaying IDD in naturally aged mice. Hence, MA holds the potential to serve as an alternative therapeutic approach for IDD.

## 1 Introduction

Over 540 million individuals worldwide suffer from low back pain (LBP), making it one of the most pressing public health issues today. LBP significantly burdens families and elderly individuals and leads to increased rates of disability (Hartvigsen et al., 2018; Knezevic et al., 2021). While the exact cause of LBP is not fully understood, intervertebral disc degeneration (IDD) is a major contributing factor, often leading to severe systemic disability (Mohd Isa et al., 2022). IDD refers to a series of pathological changes that occur within the intervertebral disc. It is characterized by the destruction of the extracellular matrix (ECM), including a reduction in proteoglycans and collagen fibers, which leads to a decrease in disc height, a decline in water content, and an impairment of the normal biomechanical function of the intervertebral disc (Li et al., 2022). The etiology of IDD is multifactorial. Aging is a prominent factor. As people age, cells within the intervertebral disc, such as chondrocytes and nucleus pulposus cells, will experience senescence, and their ability to maintain the integrity of the ECM will also decline. In addition, the development of IDD may be exacerbated by inflammatory responses, injuries, obesity, smoking, and abnormal mechanical loading (Silwal et al., 2023). Current treatment methods for IDD, including drug therapy, conservative treatment, and surgical treatment, can only provide short-term pain relief and cannot halt the degenerative process (Paesold et al., 2007; Dowdell et al., 2017). Therefore, it is crucial to develop effective therapeutic drugs and explore the potential mechanisms for the prevention and treatment of IDD.

Increasing evidence suggests that vertebral osteoporosis and endplate osteochondral remodeling are critical in IDD progression (Li et al., 2023). The intervertebral disc is nonvascularized, relying on the cartilaginous endplate—a hyaline cartilage composed of chondrocytes and ECM—for nutrient exchange and metabolism between the disc and vertebral bone (Feng et al., 2016). Bone marrow contact channels in cartilaginous endplates facilitate nutrient delivery to the intervertebral disc (IVD) and the removal of metabolic waste through fluid flow and diffusion during cyclic loading (Holm et al., 1981; Mattei, 2013). During spinal degeneration, cartilage endplates undergo chondrocyte hypertrophy and calcification and are resorbed by osteoclasts to form bony porous structures. This can lead to abnormal peri-intervertebral disc innervation, exacerbating IDD and increasing the incidence of LBP (Ling et al., 2023). Similarly osteoporosis plays an important role in IDD. Bhadouria et al. (2022) demonstrated that raloxifene, an antiosteoporotic medication, alleviated degenerative disc pain in postmenopausal women affected by aging, biological sex, and decreased estrogen levels. Alendronate has been shown to increase bone mass and slow IDD progression in ovariectomized rats (Luo et al., 2013). Liu et al. reported that calcitoninogen enhanced bone strength and density, which was associated with delayed IDD in ovariectomized rats (Liu et al., 2015; Dos Santos et al., 2022). While some clinical studies have reported a negative link between osteoporosis and IDD, these conditions often coexist, suggesting interconnected developmental processes (Nanjo et al., 2003; Mattei, 2013).

Previous research has demonstrated that myristic acid (MA) (hereinafter abbreviated as MA, with the molecular formula CH3(CH2)12COOH) represents a type of saturated fatty acid. It is predominantly present in nutmeg oil, coconut oil, palm kernel oil, milk fat, and cow’s milk, and serves as a key ingredient in the production of numerous food products (Dos Santos et al., 2022). Previous studies have shown that MA enhances spatial memory in mice and may prevent hippocampal aging induced by GABAergic signaling (Shang et al., 2022). Myristoleic acid, an isomer of MA, has been shown to suppress Receptor Activator of Nuclear Factor Kappa-B Ligand (RANKL) activation, reducing osteoclastogenesis and bone resorption (Kwon et al., 2015). These findings suggest that MA might have therapeutic potential in age-related conditions such as osteoporosis and osteoarthritis. However, there are no *in vivo* data on the impact of MA on IDD in naturally aged mice. This study aimed to determine whether MA could delay the IDD of naturally aged mice by slowing down the endplate osteochondral remodeling and vertebral osteoporosis. Through *in vitro* cellular experiments and a naturally aged mouse model, we investigated the effects of MA on IDD, thus laying a foundation for its potential clinical application.

## 2 Materials and methods

### 2.1 Animals and designs

Three-month-old and 22-month-old C57BL/6 mice and SD rats were obtained from the Laboratory Animal Center of Guangzhou University of Traditional Chinese Medicine (Animal Production Certificate # (GD) 20180034). The mice and rats complied with the standards for drinking tap water and rodent feed (product performance standard: GB14924.3-2010). All animal experiments were approved by the Ethics Committee of the First Affiliated Hospital of Guangzhou University of Chinese Medicine. (license number: TCMF1-2021026). We purchased 3-month-old young mice (3 M) and 22-month-old mice (22 M). The 22-month-old mice were randomly divided into two groups: the 22-month-old group (22 M, with an equal amount of solvent injected intraperitoneally) and the MA group (MA + 22 M, with 2 mg/kg MA injected intraperitoneally). Intraperitoneal injection was administered for 2 months, and the concentration of MA was determined according to a previous study by our team (Shang et al., 2022). Mouse sampling was performed after 2 months, and L3-L5 segmental spines were collected for subsequent experiments (Ling et al., 2023).

### 2.2 Microcomputed tomography analyses

The vertebrae of segments L3-L5 were scanned after paraformaldehyde fixation via a micro-CT imaging system at 80 kV, 100 μA current and 12 μm resolution. The acquired images were corrected, analyzed and reconstructed via Data Viewer, CTAn v1.14 and CTvox v3.0 software. The parameters evaluated and recorded included the bone volume fraction (BV/TV), trabecular thickness (Tb.Th), trabecular number (Tb.N), and trabecular separation (Tb.Sp). The three-dimensional structural parameters of the analyzed endplates included the number of closed pores [Po. N (cl)], total pore volume [Po. V (tot)], overall porosity, and total tissue volume (TV).

### 2.3 Histology and immunohistochemistry

After the vertebrae were fixed with 4% paraformaldehyde for 72 h, they were decalcified in 0.5 M ethylenediaminetetraacetic acid (EDTA) for one month at room temperature, followed by paraffin embedding. After decalcification, the coronal surfaces of the L3 to L5 vertebrae were cut into longitudinal sections 4 μm thick, and we simultaneously extracted the five viscera of the mice to make the above sections for staining. Hematoxylin‒eosin (H&E) staining solution, safranin-O green staining solution, masson staining solution, Tartrate—Resistant Acid Phosphatase (TRAP) staining solution, and specific stains of Aggrecan (Beyotime, AF6126) and Collagen II (Beyotime, AF1078) were used in accordance with standard procedures for the observation of various cellular components. All images were captured with a digital pathology scanner (KF-FL-005, Ningbo Jiangfeng Bioinformatics Co., Ltd.) instrument. The degree of disc degeneration was evaluated in HE-stained sections. The scoring system is capable of analyzing key histopathological features, including the nucleus pulposus, annulus fibrosus, cartilaginous endplates, and annulus fibrosus/nucleus pulposus/cartilaginous endplate border region (Melgoza et al., 2021). Each image was independently scored by two blinded evaluators and then averaged for further analysis.

### 2.4 Cell culture

Primary rat endplate chondrocytes were collected from 6-week-old SD rats (Charles River, Guangdong Viton Lihua Laboratory Animal Technology Co., Ltd.). Peroxyacetic acid was used to disinfect the beheaded rats. After being aseptically isolated, the endplate cartilage in the rat tail intervertebral disc was rinsed three times with phosphate-buffered saline (PBS). The cartilage endplates subsequently underwent 4 h of constant temperature shaking at 37°C in media containing 0.25% type II collagenase (Biosharp, #BS164). The cartilage endplate cells were digested and then suspended in DMEM/F-12 media [15% fetal bovine serum (FBS), 1% streptomycin/penicillin]. The medium was then changed every 3–4 days while the cells were cultured at 37°C and 5% CO^2^. We looked at the cells under a microscope. After using 1:2–3 passages to develop the cells to 80% confluence, the P2–P3 generations were eliminated for use in later cell investigations.

### 2.5 Immunofluorescence and β-galactosidase staining

The cartilage endplate cells were plated in 24-well plates at a density of 10^4^/well, and the cells were cultured in a 37°C cell culture incubator for 1 day to allow the cells to adhere to the wall. After intervention, the cells were washed with PBS, fixed with paraformaldehyde at room temperature and permeabilized with 0.3% Triton X-100. After washing, the cells were sealed with immunostaining sealing solution, and after sealing, the sealing solution was aspirated, and the primary antibody was added and incubated overnight. The next day, the primary antibody was collected, and the cells were washed with PBS. Then, the secondary antibody was added, and the mixture was incubated at room temperature and protected from light. After incubation, the cells were washed with PBS and finally labeled with DAPI (Beyotime, P0131) contained in anti-fluorescence quenching blocking solution for 10 min. We performed galactose staining (Solarbio) to validate the phenotypic experiments of MA for delayed aging. F-actin ring staining was also performed to visualize osteoblast formation, and the RAW264.7 cells were washed as described above, treated with iFluor™ 488 phalloidin for 2 h and treated with DAPI for 10 min. Finally, the stained cells were photographed via a BioTek Cytation C10 confocal microplate imaging detection system.

### 2.6 Bioinformatics analysis

We searched the GEO database for gene expression profiles related to IDD, osteoporosis, and cartilage endplate degeneration via the keywords “disc degeneration”, “osteoporosis”, and “endplate osteochondral degeneration.” We chose the GEO datasets numbered GSE124272, GSE35958, and GSE153761, and the series matrix files provided by the data providers contained the normalized data processed by the MAS5 algorithm. We performed DEG analysis on the datasets of the three diseases to validate shared and unique genes among the three diseases. The R package “limma” was used to identify DEGs between the case and control groups, with a cutoff value of *p* < 0.05. Hierarchical clustering heatmaps and volcano maps were used to reveal the expression patterns of these DEGs. GO and KEGG pathway enrichment analyses were performed to further elucidate the biological functions of the genes associated with the hub genes. The 3D structure of the monomeric drug MA was obtained from PubChem (http://pubchem.ncbi.nlm.nih.gov) and converted to MOL2 file format via the Open Babel Toolkit (version 2.4.1). In the PDB format of the RCSB Protein Data Bank (http://www.rcsb.org/), AutoDock software was used to optimize the protein structures for molecular docking of myristic acid with ACAN (PDB: 3B8Z), MMP3 (PDB: 4DPE), MMP9 (PDB: 1L6J), and p21 (PDB: 5P21). docking. Finally, the docking results were visualized via PyMOL (version 2.4.1).

### 2.7 Cell viability assay

Using a Cell Counting Kit-8 (CCK-8, Glpbio, GK10001) test, the cytotoxic effects of TBHP and MA on primary chondrocytes were assessed in accordance with the manufacturer’s instructions. A total of 10,000 cells per well of 96-well plates were used to inoculate the cells. Next, the cells were treated for 24 h, 48 h, or 72 h with various dosages of MA or for 2 hours with various concentrations of TBHP. Each well was then filled with CCK-8 reagent, and the cells were cultured for an additional 2 hours. Then, for 2 hours, the cells were treated with TBHP. TBHP was subsequently applied to the cells for 2 hours. Then, for 2 hours, the cells were treated with TBHP. Ultimately, an enzyme marker at 450 nm was used for the detection of the absorbance of each well.

### 2.8 Quantitative RT-qPCR

Using TRIzol reagent, total RNA from endplate chondrocytes was extracted, and a cDNA synthesis kit (EZBioscience, #A0010CGQ) was used for reverse transcription. 2x Color SYBR Green qPCR Master Mix (EZBioscience, #A0012-R2) was used for reverse transcription quantitative polymerase chain reaction (RT-qPCR). QuantStudioTM real-time PCR software was used to carry out the quantitative analysis. The reference genes for quantitative analysis were GAPDH and β-actin. Table 1 displays the primer sequences for the target genes utilized in this investigation.

**TABLE 1:** Lists the sequences of primers used in RT-qPCR.

Gene	Forward primer (5′-3′)	Reverse primers (5′-3′)	TM(°C)
Aggrecan	CGCTACTCGCTGACCTTT	GCTCATAGCCTGCTTCGT	60
Collagen II	GAG​TGG​AAG​AGC​GGA​GAC​TAC​TG	CTC​CAT​GTT​GCA​GAA​GAC​TTT​CA	60
SOX-9	TCC​CCG​CAA​CAG​ATC​TCC​TA	AGC​TGT​GTG​TAG​ACG​GGT​TG	60
MMP3	TTT​GGC​CGT​CTC​TTC​CAT​CC	GCA​TCG​ATC​TTC​TGG​ACG​GT	60
MMP9	CTA​CAC​GGA​GCA​TGG​CAA​CGG	TGG​TGC​AGG​CAG​AGT​AGG​AGT​G	60
MMP13	TCC​ATC​CCG​AGA​CCT​CAT​GT	AGC​ATC​ATC​ATA​ACT​CCA​CAC​G	60
p16	CGG​TAT​TTG​CGG​TAT​CTA​CTC​TCC	CCA​GAA​GTG​AAG​CCA​AGG​AGA​A	60
p21	CAA​AGT​ATG​CCG​TCG​TCT​GTT​C	GTC​AAA​GTT​CCA​CCG​TTC​TCG	60
p53	CCC​CTG​AAG​ACT​GGA​TAA​CTG​T	GAC​AGG​CAC​AAA​CAC​GAA​CC	60
ADAMTS-5	GGA​CCT​ACC​ACG​AAA​GCA​GAT​C	GCCGGGACACACGGAGTA	60
GAPDH	GCAAGTTCAACGGCACAG	CGC​CAG​TAG​ACT​CCA​CGA​C	60
ACP5	CAC​TCC​CAC​CCT​GAG​ATT​TGT	CAT​CGT​CTG​CAC​GGT​TCT​G	60
DC-STAMP	CGG​CGG​CCA​ATC​TAA​GGT​C	CCC​ACC​ATG​CCC​TTG​AAC​A	60
CTSK	TCA​TCC​ACT​ACC​GTG​TTT​TGC	GTG​CTC​CAC​CAT​GTC​CAT​CA	60
β-actin	AGGGAAATCGTGCGTGAC	CAT​ACC​CAA​GAA​GGA​AGG​CT	60

### 2.9 Flow cytometry

For the identification of cartilage endplate cells, we performed flow cytometry. After the cells were rinsed with PBS and 0.25% trypsin for digestion, the cell suspension was transferred to a centrifuge tube and centrifuged at 1,000 r/min for 5 min. The supernatant was discarded, the cell precipitate was collected, the cells were washed twice with PBS, the cells were counted, the cell suspension was made into 10^6^/mL EP tubes, the concentration was increased to 200 U with a FITC-conjugated fluorescent antibody (BioLegend) at a ratio of 1:100 (APC-conjugated anti-mouse/rat CD29 antibody, FITC-conjugated anti-mouse/rat CD45 antibody, and PE-conjugated anti-mouse/rat CD90), and a peer control group was established. The cells were incubated at 37°C and washed twice with PBS.

### 2.10 Statistical analysis

The experimental data were statistically analyzed using GraphPad Prism 8.0.1 software. The measurement data were represented by mean ± standard deviation ( $\bar{x}$ ±s). When comparing the results among different groups in the experiment, one-way analysis of variance (ANOVA) followed by Tukey’s multiple comparisons test was used. While when comparing the results between two groups, an independent samples *t*-test was employed. A statistically significant difference was indicated when *p* < 0.05.

## 3 Results

### 3.1 MA treatment mitigates endplate osteochondral remodeling and improves vertebral bone microstructure in aged mice

**FIGURE 1:**
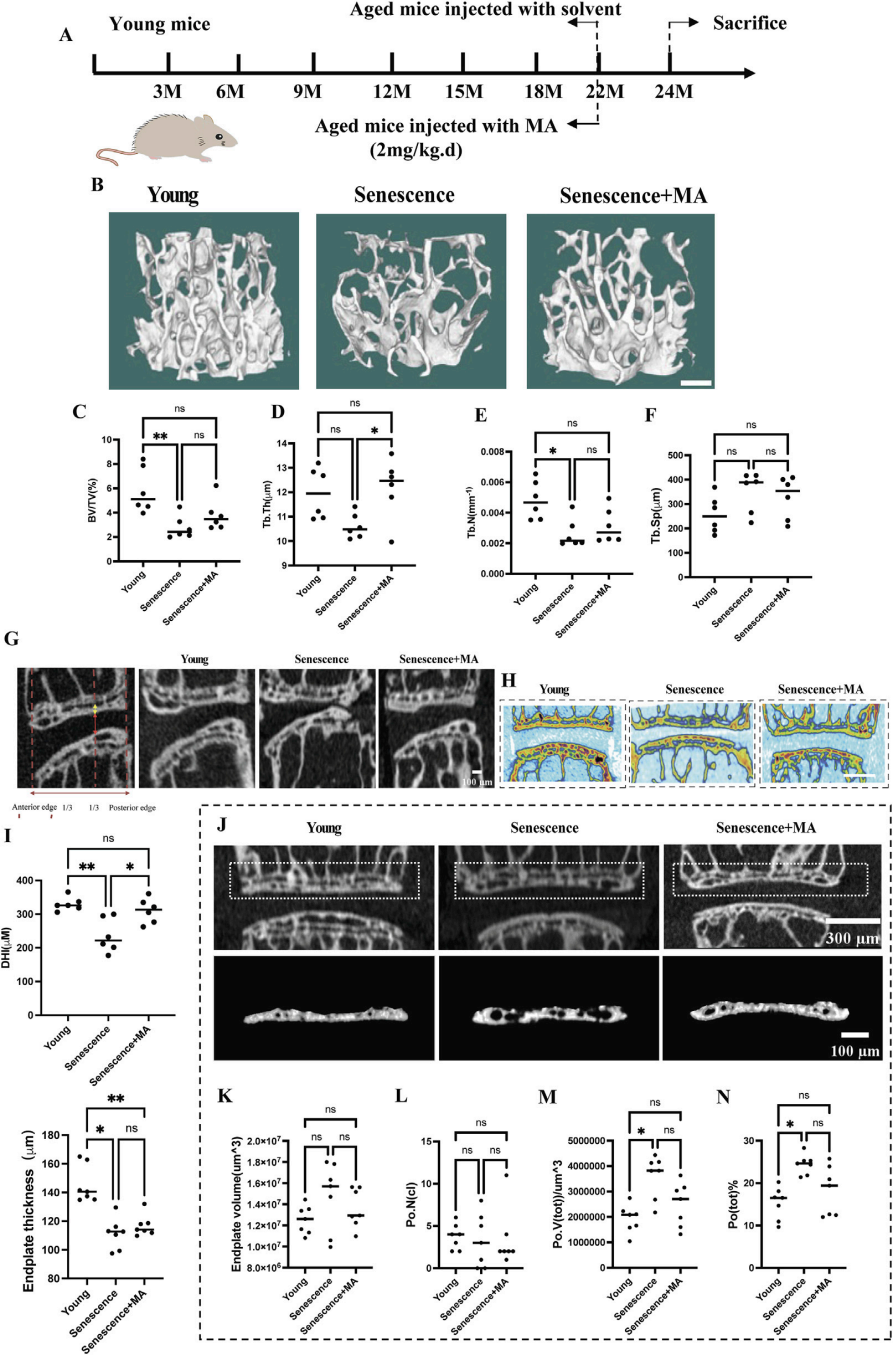
Effects of MA on intervertebral disc height, endplate thickness, and vertebral bone microstructure in aged mice. **(A)** Schematic representation of the MA treatment protocol for naturally aged mice. **(B)** Representative micro-CT images of L4 vertebrae from different groups (scale bars: 50 μm). **(C–F)** Quantitative analysis of L4 vertebrae showing bone volume/total volume (BV/TV), trabecular thickness (Tb.Th; μm), trabecular number (Tb.N; mm⁻^1^), and trabecular separation (Tb.Sp; μm). **(G–I)** Representative micro-CT scans of the L4-L5 region in the coronal and sagittal planes. The height of the nucleus pulposus (NP) is indicated by the red arrow, and the height of the cranial endplate (EP) is indicated by the yellow arrow (scale bars: 100 μm, 500 μm). **(J)** Quantitative analysis of cranial endplate thickness at L4-5 and disc height. **(K)** Representative three-dimensional reconstructions (bottom) and coronal high-resolution μCT images (top) of L4/5 endplates from each group (scale bars: 300 μm, 100 μm). **(L–N)** Quantitative analysis showing changes in the endplate volume, number of closed pores [Po. N (cl)], total pore volume [Po. V (tot)], and total porosity percentage [Po. (tot)%] across the groups. Compared with the young group, the senescent group presented increased Po. (tot)%, Po. V (tot), and Po. N (cl). Compared with no treatment, MA treatment resulted in a reduction in these parameters in senescent mice (n = 8 per group). Statistical significance: **p < 0.01, *p < 0.05, ns: not significant.

To investigate the impact of MA on endplate osteochondral remodeling during disc degeneration, we employed a model using naturally aged mice. The mice were categorized into three groups: young mice, senescent mice (treated intraperitoneally with solvent), and MA-treated mice (senescent mice treated with 2 mg/kg MA) (Figure 1A). Micro-CT analysis revealed that, compared with the young group, the senescent group presented sparser trabeculae and wider trabecular gaps. In contrast, the MA group presented a slightly denser trabecular structure and relatively reduced trabecular spacing compared with those of the senescent group. Specifically, the bone volume/total volume (BV/TV) ratio and trabecular number (Tb.N) were significantly lower, the trabecular thickness (Tb.Th) was lower, and the trabecular spacing (Tb.Sp) was greater in the senescent group than in the young group. Compared with the senescent group, MA significantly improved the Tb.Th, whereas there was a decreasing trend in the Tb. Sp and an increasing trend in the BV/TV and Tb.N after MA intervention, but these differences were not statistically significant (Figures 1B–F). We examined the micro-CT parameters of the endplates at the L4-L5 segment to evaluate the microstructure of the vertebral bone and endplates and calculated the disc height index (DHI) from L4-L5 as a surrogate marker of disc degeneration. Compared with that in the senescent group, the DHI was significantly greater in the MA treatment group, and the endplate thickness tended to increase but was not significantly different. However, DHI and endplate thickness were significantly lower in the senescent group than in the young group (Figures 1G–I). The number of closed pores [Po. N (cl)] decreased and cranial endplate volume (CEV) increased in the senescence group compared with those in the young group, but neither difference was statistically significant. The total pore volume [Po. V (tot)], and the percentage of total porosity [Po(tot)%] was significantly greater in the senescent group than in the young group. However, cranial endplate volume (CEV), total porosity percentage [Po(tot)%], and total pore volume [Po. V (tot)] tended to decrease in the MA group compared with the senescence group but was not significantly different. These results suggest that MA ameliorated vertebral space collapse in senescent mice and inhibited vertebral osteoporosis and osteochondral remodeling of the endplate to a certain degree (Figures 1J–N).

### 3.2 MA intervention ameliorates disc degeneration and restores cartilage endplate thickness in aged mice

**FIGURE 2:**
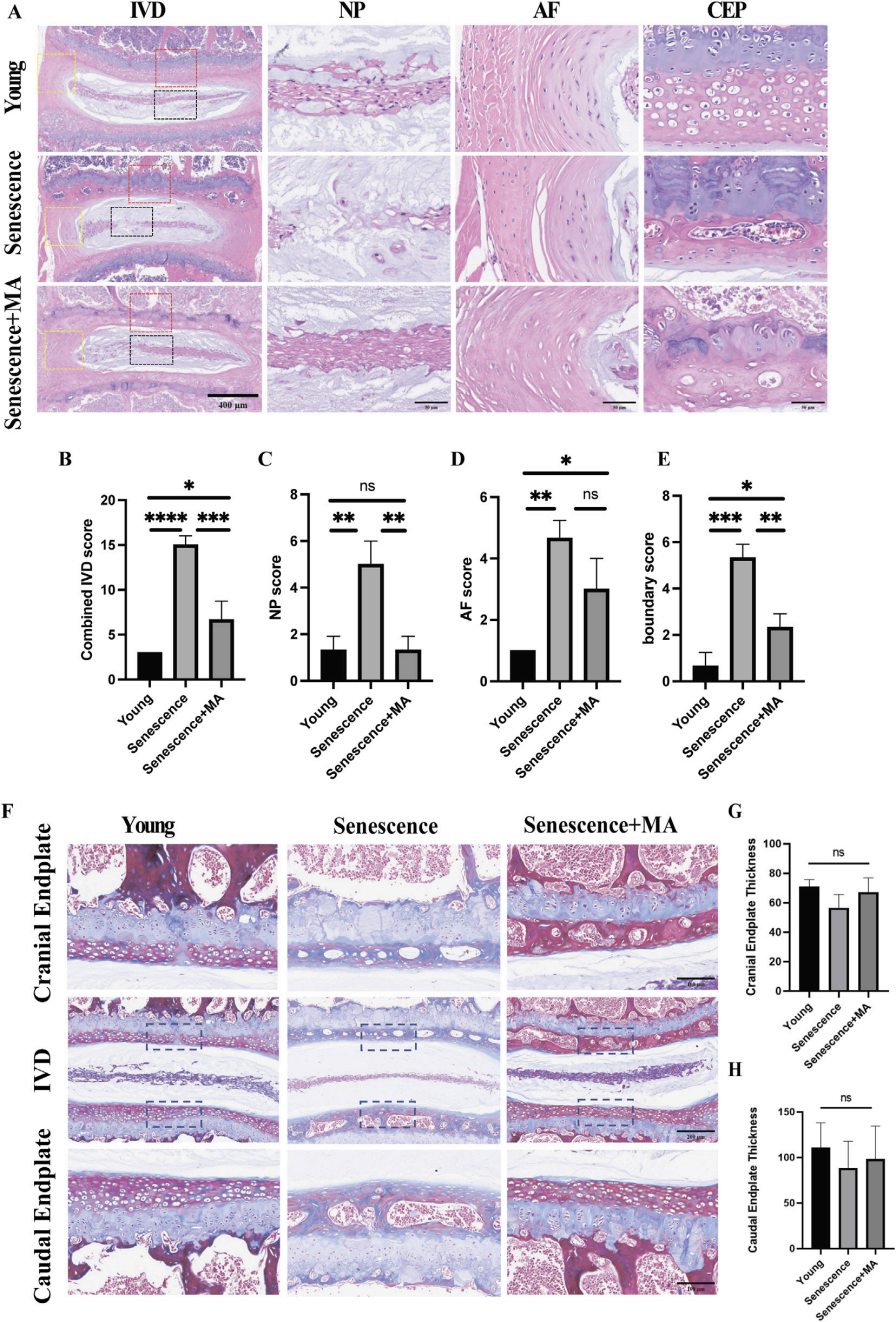
MA delays disc degeneration and restores cartilage endplate thickness in aged mice. **(A)** H&E staining results of L4‒5 segment from the mice in each group. The L4-5 intervertebral disc, the L4-5 nucleus pulposus tissue, and the annulus fibrosus tissue structure are depicted in the image (scale bars: 400 μm, 50 μm). **(B–E)** In the young, senescent, and MA groups, the sum of the IVD, NP, AF, and AF/NP/EP boundary scores is shown. IVD, intervertebral disc; EP, endplate; AF, annulus fibrosus; NP, nucleus pulposus. **(F)** Results of L4‒5 segment Masson staining in various mouse groups. The L4-5 intervertebral disc, cranial endplate, and caudal endplate are depicted in the figure (scale bars: 100 μm, 200 μm). **(G)** Examination of cranial endplate thickness in L4–5 segmental discs from the MA, senescent, and young groups. **(H)** Analysis of caudal endplate thickness in L4–5 segmental discs in the MA, senescent, and young groups (n = 3 per group). Statistical significance: *p < 0.05; **p < 0.01; ***p < 0.001; ****p < 0.0001; ns, not significant.

We performed Masson and H&E staining to evaluate disc organization across three groups of mice: young, senescent, and MA-treated senescent mice. In young mice, the intervertebral discs presented a well-preserved structure, with distinct nucleus NP tissue boundaries surrounded by a dense extracellular matrix (Figure 2A). However, naturally aged mice displayed pronounced degenerative changes, including fissures, significant loss of extracellular matrix in the cartilaginous endplates, and disrupted boundaries between the endplates and the NP. Notably, the NP tissue of aged mice presented a marked decrease in cell number, disorganized architecture, visible scarring, and increased blending of fibrous tissue into the AF. In contrast, MA-treated senescent mice demonstrated considerable structural improvements. The intervertebral discs of this group presented clearer tissue boundaries and more uniformly distributed cells in the NP, with a morphology closely resembling that of young mice. The AF fibers and the boundaries between the AF and NP tissues were also distinctly demarcated. Importantly, the number of NP cells was significantly greater in the MA-treated group than in the senescent group, indicating reduced cell senescence and death. Quantitative histopathological scoring of the intervertebral discs, which included assessments of the NP, AF, and AF/NP/EP boundary regions, revealed that the senescent group had significantly higher scores than did the young group, reflecting more severe degeneration. Conversely, the MA-treated group had lower scores than did the senescent group did, indicating the amelioration of degenerative changes (Figures 2B–E). Compared with the young group, the senescent group presented significantly fewer collagen fibers, reduced cartilage endplate thickness, and a more disorganized disc structure (Figure 2F). Compared with those in the senescent group, there was a tendency toward increased collagen fiber and cartilage endplate thickness on the cranial and caudal sides after treatment with MA, but the difference was not statistically significant (Figures 2G, H). In conclusion, MA was effective in alleviating disc degeneration, enhancing collagen fiber deposition, and restoring cartilage endplate thickness in naturally aged mice. These findings suggest that MA has a potential therapeutic role in counteracting age-related disc degeneration by protecting the structural and cellular integrity of the intervertebral disc.

### 3.3 MA inhibits cartilage endplate remodeling and reduces nucleus pulposus cell destruction in naturally aged mice

**FIGURE 3:**
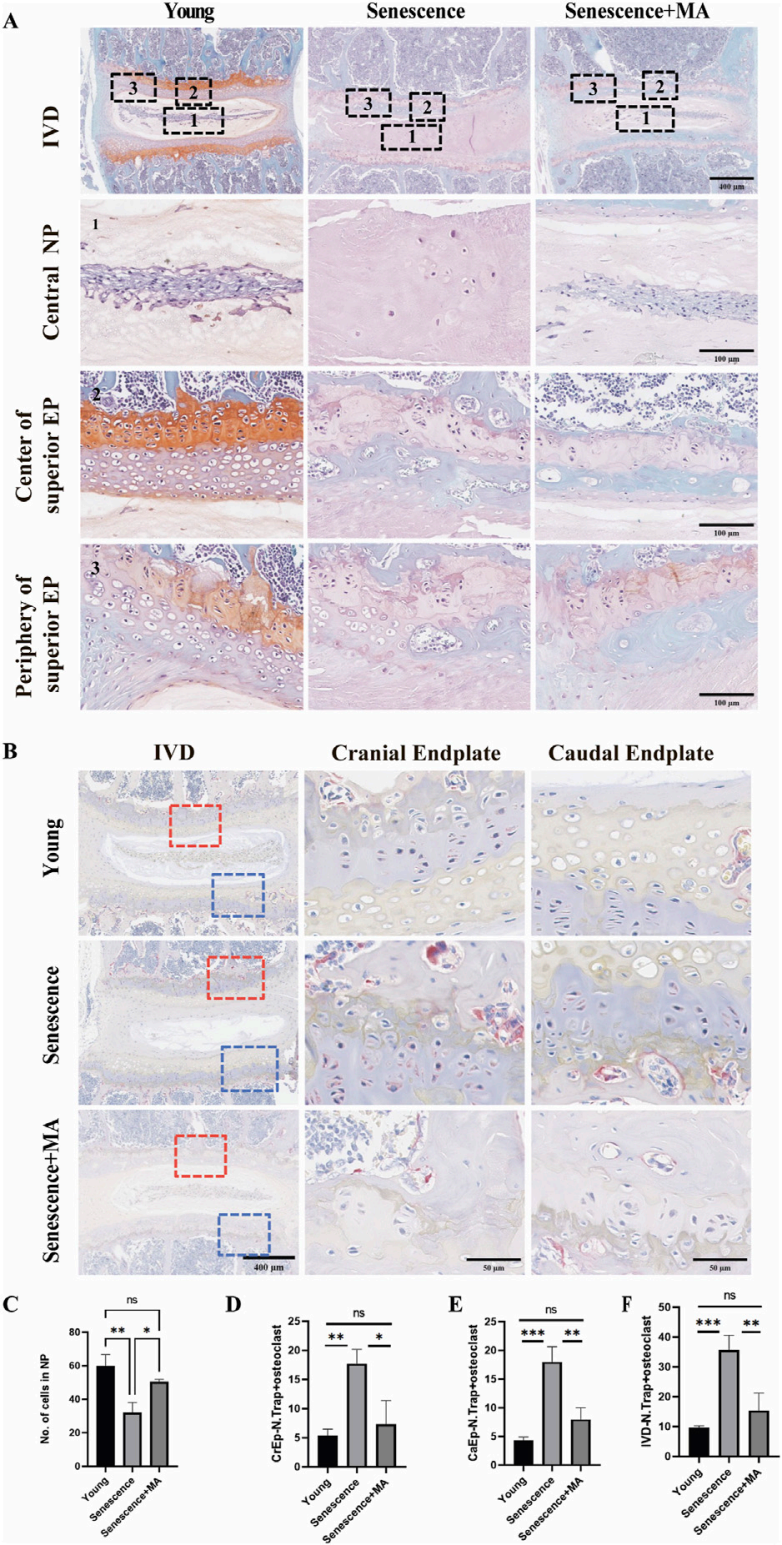
MA inhibits the remodeling of cartilage endplates in senescent mice. **(A)** Results of L4‒5 segment stained with safranin-O green in various groups of mice. The L4-5 intervertebral disc, L4-5 nucleus pulposus, superior EP center, and superior EP periphery are depicted in the figure (scale bars: 400 μm, 100 μm). **(B)** The endplates of the young and MA groups presented a low density of TRAP-positive cells, which were primarily observed on the surface of the trabeculae of the subchondral bone. Nevertheless, in the Senescence group, the subchondral bone and endplate had a much greater frequency of TRAP-positive cells, indicating a decrease in osteoclast activity following MA therapy (scale bars, 400 μm, 50 μm). **(C)** Statistical examination of the nucleus pulposus cell counts of the young, senescent, and MA groups. **(D–F)** Quantitative evaluation of TRAP-positive cells in the IVD, CrEP, and CaEP groups (n = 3 per group). Statistical significance: *p < 0.05, **p < 0.01. IVD intervertebral disc, CrEP cranial endplate, CaEP caudal endplate.

To evaluate the impact of MA on cartilage endplates remodeling in senescent mice, we performed TRAP staining and Safranin-O green staining. Compared with young mice, the senescent group presented a greater number of osteoclasts in both the upper and lower cartilage endplates. This increased osteoclast number was associated with more pronounced cartilage remodeling and endplate degeneration, underscoring the significant influence of aging on bone and cartilage dynamics. Compared with that in the senescent group, the number of osteoclasts within the cartilage endplates in the MA-treated group was notably lower (Figures 3B, D–F). This reduction indicates a decrease in bone resorption activity following MA treatment. Additionally, the Safranin-O green staining results suggested that the extent of remodeled cavities in the cartilage endplates was significantly lower in the MA group than in the senescent group. The number of NP cells within the intervertebral discs significantly increased, and the cartilage area at the endplate locations also increased after MA intervention (Figures 3A, C). These findings suggest that MA treatment effectively mitigates cartilage endplate remodeling and delays the loss of NP cells in naturally aged mice. By reducing osteoclast activity and preserving cartilage structure, MA helps maintain the integrity of intervertebral discs and prevents age-related degeneration.

### 3.4 MA alters matrix composition in NP tissue and demonstrates safety in senescent mice

**FIGURE 4:**
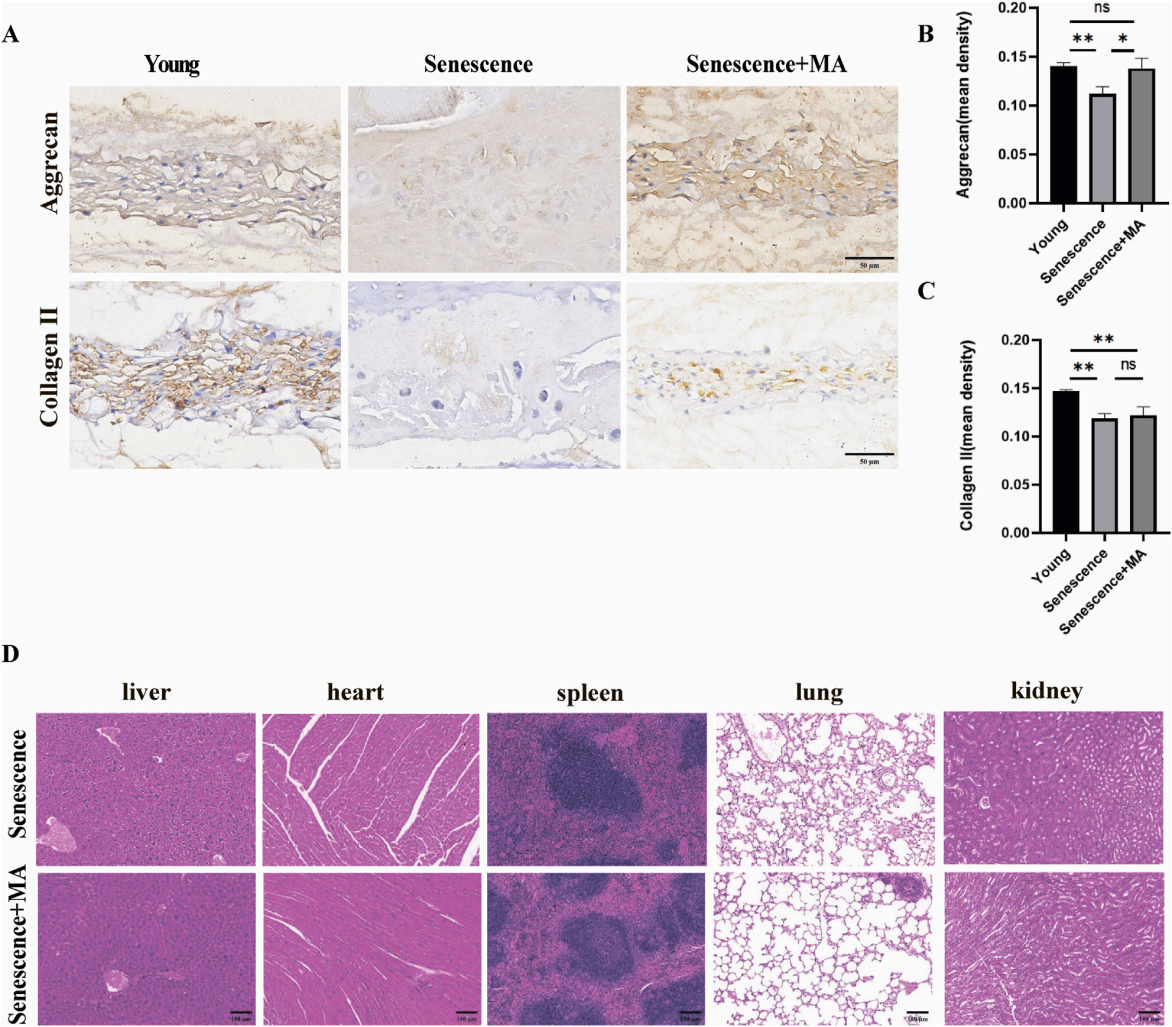
MA can delay disc degeneration by altering the matrix composition of NP tissue. **(A)** Aggrecan and Collagen II immunohistochemical results in the NP tissues of the MA, senescent, and young groups (scale bars, 50 μm). **(B, C)** The immunohistochemical results demonstrated that the MA group had higher levels of positive staining and higher expression of the Collagen II and Aggrecan proteins than did the senescence group. **(D)** Results of HE staining for the MA and senescence groups. The histologic features of the stained sections of the liver, heart, spleen, lung, and kidney were identical between the senescent and MA groups (scale bars, 100 μm). The sample size was n = 3 per group. Statistical significance: *p < 0.05, **p < 0.01; ns, not significant.

To evaluate the impact of MA on matrix metabolism within the NP tissue, we performed immunohistochemical analysis to compare the expression of Aggrecan and Collagen II among the young, senescent, and MA-treated groups. The NP, which comprises primarily gelatinous components, is a critical functional element of intervertebral discs. Degradation of the NP is a key step in the progression of IDD. Compared with those in the young group, the NP in the senescent group displayed reduced staining intensity and lower immunoreactivity for Aggrecan and Collagen II, indicating high levels of both proteins. Conversely, the MA-treated group presented significantly greater staining intensities for Aggrecan and Collagen II in the NP tissue than did the senescent group (Figures 4A–C). These findings suggest that MA can delay disc degeneration by altering the composition of matrix components within the NP. Additionally, we performed H&E staining to assess the histological structure of five major organs—liver, heart, spleen, lung, and kidney—in the MA-treated and senescent groups. The results revealed no significant differences in the histological structures of these organs between the two groups, indicating that MA administration was not pharmacologically harmful to senescent mice (Figure 4D).

### 3.5 Bioinformatics analysis reveals interconnections between intervertebral disc degeneration, osteoporosis, and endplate osteochondral degeneration linked to cellular aging processes

**FIGURE 5:**
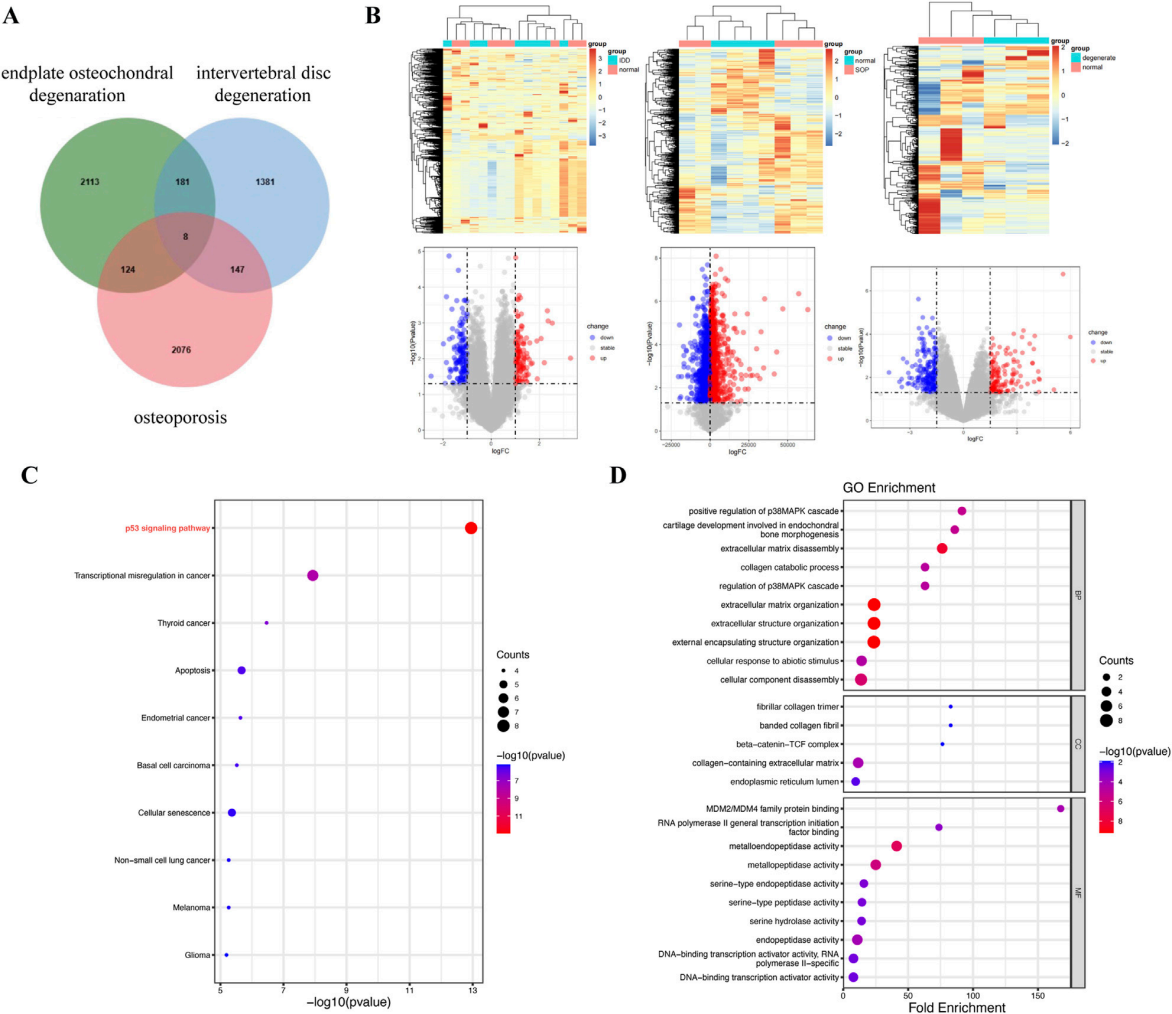
Bioinformatics analysis of three diseases revealed the following: disc degeneration, osteoporosis, and endplate osteochondral degeneration. **(A)** Venn diagram of overlap (between endplate osteochondral degeneration, osteoporosis and IDD targets). **(B)** Hierarchical clustered heatmaps and volcano maps representing differential gene expression between the normal and model groups in three diseases. **(C)** KEGG pathway analysis of intersecting genes. **(D)** GO functional enrichment analysis of intersecting genes.

Bioinformatics analyses revealed eight intersecting genes common to IDD, endplate osteochondral degeneration, and osteoporosis (Figure 5A). The differential gene expression profiles between the normal and model groups for each of these three conditions were visualized through hierarchical clustered heatmaps and volcano plots (Figure 5B). KEGG pathway analysis revealed that the p53 signaling pathway was the most significantly enriched pathway among the intersecting genes (Figure 5C). This pathway is known to play a critical role in regulating cellular senescence and apoptosis. Other significantly enriched pathways included those involved in the dysregulation of transcription in various cancers, such as thyroid cancer, endometrial cancer, basal cell carcinoma, and non-small cell lung cancer, as well as those involved in cellular senescence. The GO functional enrichment analysis further revealed that the intersecting genes are involved primarily in chondrogenesis, cartilage morphogenesis, extracellular matrix degradation, and collagen catabolic processes (Figure 5D). These biological processes are intrinsically linked to the pathophysiology of disc degeneration, cartilage endplate degeneration, and osteoporosis. Our focus on the senescence process of endplate chondrocytes highlighted the involvement of the p53 signaling pathway in the senescence metabolic processes of these cells. These findings underscore the interconnected nature of these diseases and their associations with cellular aging mechanisms.

### 3.6 *In vitro* confirmation of the biological effects of MA on endplate chondrocytes

**FIGURE 6:**
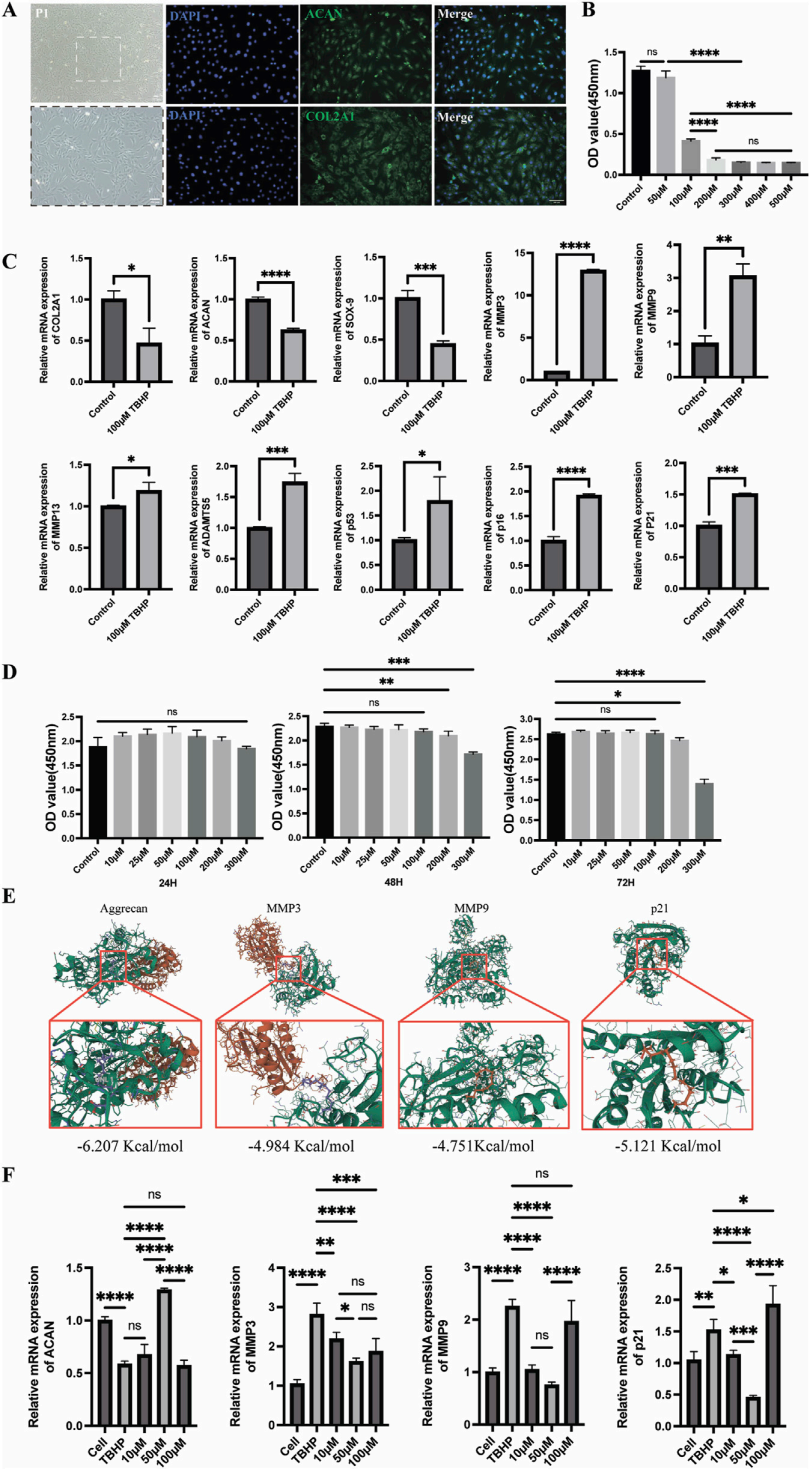
Senescent endplate chondrocytes are shielded by MA. **(A)** Collagen II (COL2A1) and Aggrecan (ACAN) immunofluorescence analysis of endplate chondrocytes (scale bars, 100 μm). **(B)** The ideal TBHP concentration for establishing an endplate chondrocytes senescence model was determined via the cholecystokinin octapeptide (CCK8) test. **(C)** Real-time qPCR demonstrated that endplate chondrocytes treated with 100 μmol/L TBHP for 2 hours were far more likely to experience endplate chondrocytes senescence. **(D)** CCK-8 was utilized to determine the right intervention concentration for MA as well as the intervention duration. **(E)** Molecular docking diagram of MA with Aggrecan, MMP3, MMP9, and p21. **(F)** RT-qPCR verified that MA delayed endplate chondrocytes senescence. The sample size was n = 3 per group. Statistical significance: *p < 0.05, **p < 0.01, ***p < 0.001; ****p < 0.0001; ns, not significant.

**FIGURE 7:**
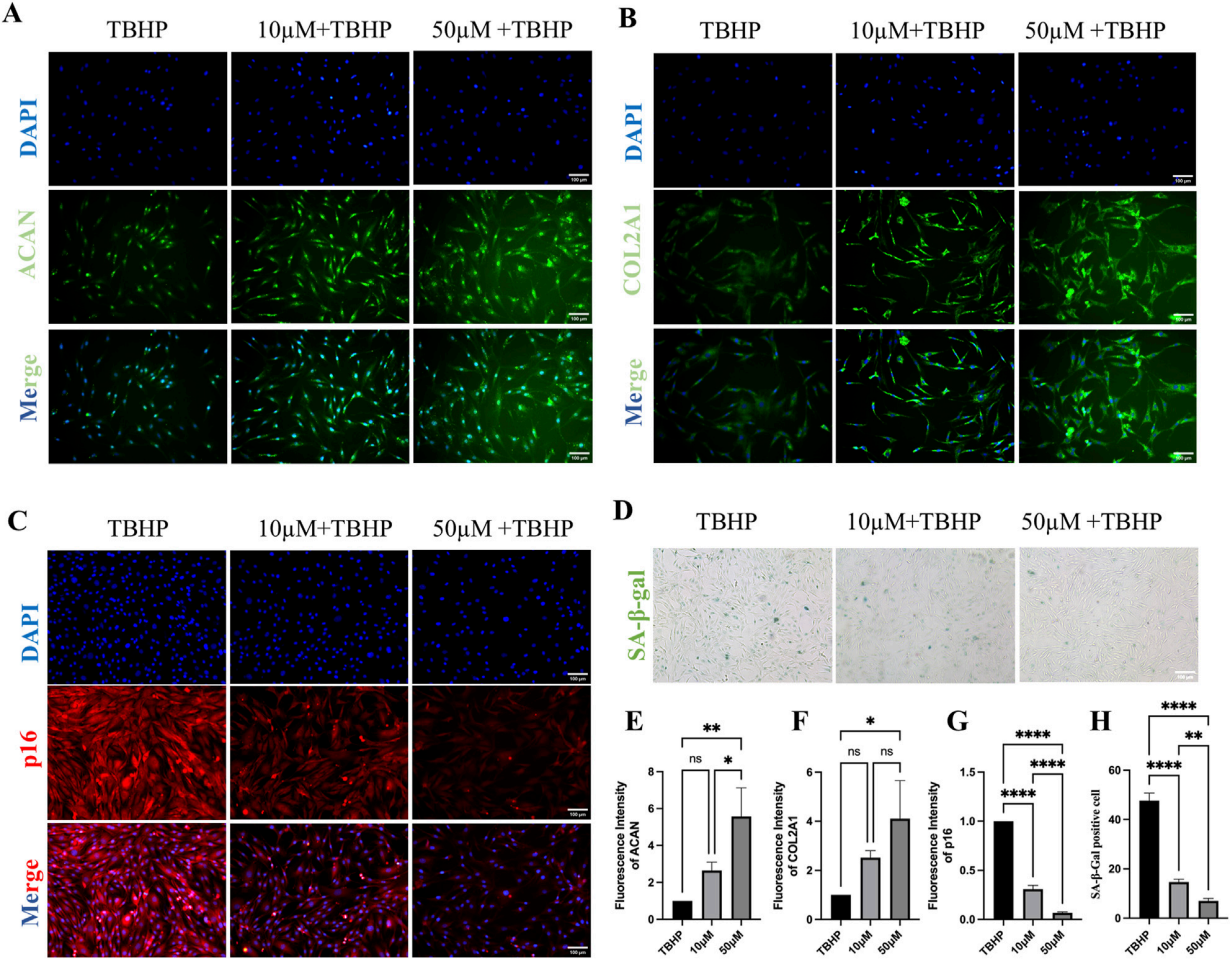
Senescent endplate chondrocytes are shielded by MA. **(A)** Aggrecan (ACAN) fluorescence staining of endplate chondrocytes. (scale bars, 100 μm). **(B)** Collagen II (COL2A1) fluorescence staining of endplate chondrocytes. (scale bars, 100 μm). **(C)** p16 fluorescence staining of endplate chondrocytes. (scale bars, 100 μm). **(D)** Effects of SA-β-gal staining on the senescence phenotype of endplate chondrocytes. (scale bars, 100 μm). **(E–H)** IF and the results of the quantitative analysis of SA-β-gal staining. The sample size was n = 3 per group. Statistical significance: *p < 0.05, **p < 0.01, ***p < 0.001; ****p < 0.0001; ns, not significant.

To validate the protective effect of MA on senescent endplate chondrocytes, we conducted a series of *in vitro* cellular assays. First, endplate chondrocytes were isolated from rats and identified via immunofluorescence. The fluorescence data confirmed the significant expression of Collagen II (COL2A1) and Aggrecan (ACAN), confirming that the isolated cells were endplate chondrocytes (Figure 6A). We also performed flow cytometric characterization, and the results indicated that the screened endplate chondrocytes barely expressed CD45 and highly expressed CD29 and CD90, suggesting that the cells we extracted were endplate chondrocytes (Supplementary Figure S1). To induce senescence in endplate chondrocytes, we exposed them to various concentrations of TBHP for 2 hours. A CCK8 assay revealed that 50 μmol/L TBHP did not significantly affect cell viability compared with that of the control group. However, exposure to other concentrations of TBHP significantly reduced the cellular activity (Figure 6B). Consequently, a concentration of 100 μmol/L TBHP for a duration of 2 h was selected for subsequent experiments. RT-qPCR analysis confirmed the establishment of the endplate chondrocytes senescence model. Compared with the control group, the model group presented significantly greater expression of the p53, p21, p16, MMP-3, MMP-9, MMP-13, and ADAMTS-5 genes and lower expression of the Aggrecan, Collagen II, and SOX-9 genes (Figure 6C). The impact of MA on endplate chondrocytes was evaluated via the CCK-8 assay. The results indicated that endplate chondrocytes viability was unaffected by MA at doses of 0, 10, 25, 50, 100, 200, and 300 μM for 24 h. Additionally, cell viability remained unchanged at these concentrations for 48 and 72 h, provided that the dose did not exceed 100 μM (Figure 6D). The RT-qPCR results demonstrated that 10 and 50 μmol/L MA significantly downregulated the expression of MMP-3, MMP-9, and the senescence marker p21 in senescent endplate chondrocytes (Figure 6E). The SA-β-gal staining results indicated that MA was able to delay the senescence of endplate chondrocytes (Figures 7D, H). The IF results indicated that MA significantly upregulated the expression of Aggrecan and Collagen II and downregulated the expression of the senescence marker p16 in senescent endplate chondrocytes (Figures 7A–C, E–G). Molecular docking analysis revealed that MA had strong binding affinities for Aggrecan (binding energy: −6.027 kcal/mol), MMP3 (binding energy: −4.984 kcal/mol), MMP9 (binding energy: −4.751 kcal/mol), and p21 (binding energy: −5.121 kcal/mol) (Figure 6F). In summary, MA ameliorates endplate chondrocyte senescence.

### 3.7 *In vitro* validation that MA inhibits osteoblastic differentiation

**FIGURE 8:**
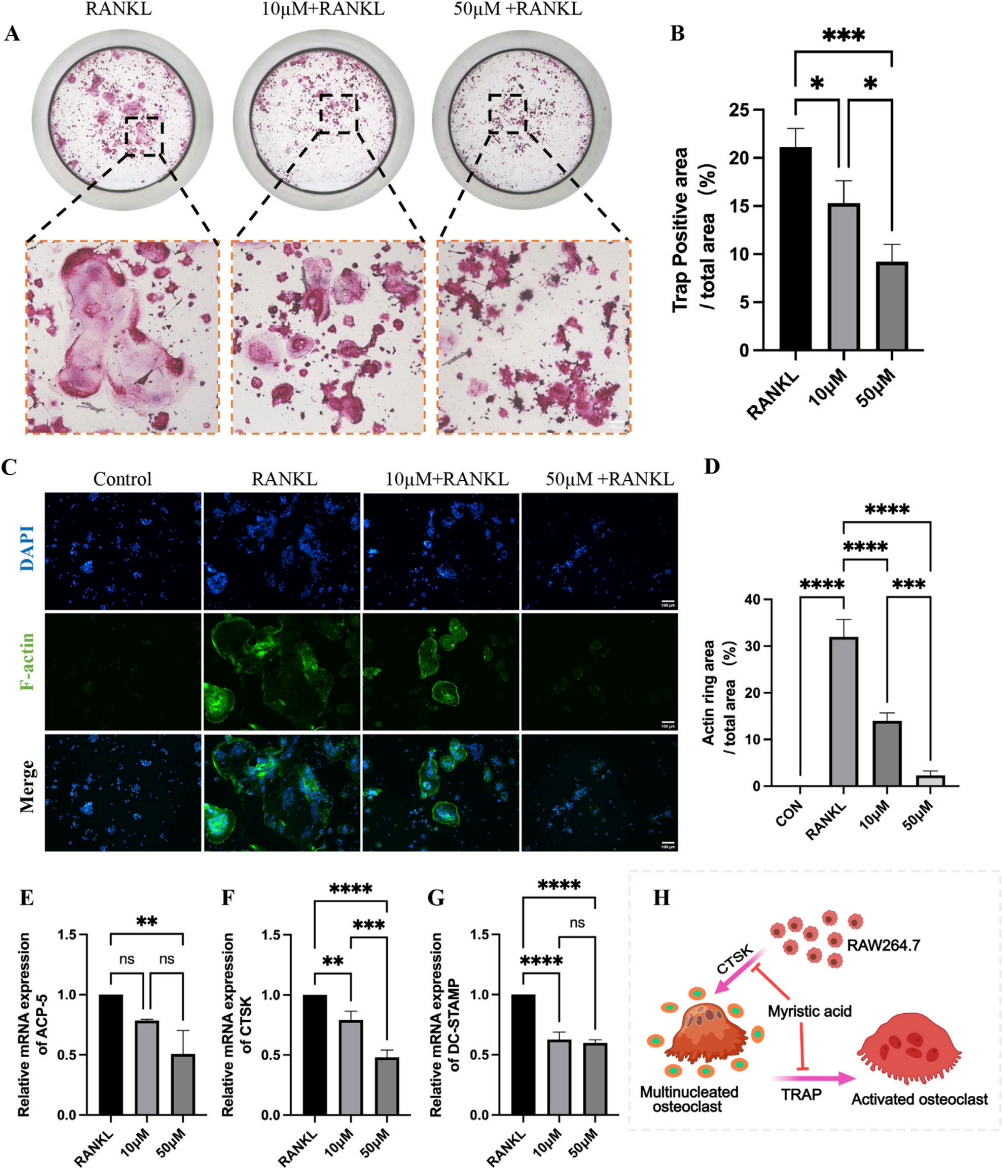
*In vitro* validation that MA inhibits osteoblastic differentiation. **(A)** Representative images of tartrate-resistant acid phosphatase (TRAP) staining. (scale bars, 100 μm). **(B)** Quantification of the TRAP-positive areas (n = 3). **(C)** Representative images of the F-actin staining assay. (scale bars, 100 μm). **(D)** Quantitation of F-actin ring areas (n = 3). **(E–G)** Real-time qPCR confirmed that MA significantly downregulated the expression of osteoclast markers. **(H)** Pattern diagram of MA inhibition of RA264.7 differentiation into osteoblasts. The sample size was n = 3 per group. Statistical significance: *p < 0.05, **p < 0.01, ***p < 0.001; ****p < 0.0001; ns, not significant.

To validate the ameliorative effect of MA on osteoporosis in a multifaceted way, we used RAW264.7 cells to perform a series of cellular experiments. Compared with that in the RANKL group, the average positive area of osteoclasts in the MA drug group was significantly reduced (Figures 8A, B), and MA significantly inhibited the osteoclastogenesis of RAW264.7 cells. F-actin staining revealed that the osteoclast formation of RAW264.7 cells in the MA group was significantly affected, and the average area of the osteoclast rings was significantly decreased (Figures 8C, D). The RT-qPCR results revealed that MA significantly downregulated the expression of the osteoblast markers ACP-5, CTSK, and DC-STAMP. Taken together, MA was able to inhibit osteoblastic differentiation at the cellular level.

## 4 Discussion

Intervertebral disc degeneration (IDD) describes the physiopathological process of disc aging and natural degradation and serves as the pathological foundation for numerous spine-related disorders (Silagi et al., 2018). IDD can lead to low back pain (LBP) and symptoms of nerve root or spinal cord compression, including vertebral instability, disc herniation, and spinal stenosis (Morris et al., 2021). In the aged population, osteoporosis and disc degeneration are highly prevalent and frequently cooccur, suggesting a possible relationship between these two conditions (Wáng, 2018). To explore this connection, we assessed the effects of Myristic acid (MA) on intervertebral disc (IVD) histology, endplate microstructure and porosity, and vertebral bone microstructure in naturally aged mice. This study is the first to show that MA, a saturated fatty acid, inhibits cartilage endplates remodeling and vertebral osteoporosis, thereby delaying disc degeneration and extracellular matrix (ECM) degradation during IDD. By preserving the anatomical and functional integrity of the vertebral bodies and endplates, MA helps maintain the patency of the nutritional channels of the intervertebral disc. Furthermore, MA regulates ECM metabolism in the NP, safeguarding the IVD structure and function.

Intervertebral discs begin to degenerate early in life and progressively worsen with age (Rider et al., 2019). In this investigation, we employed a naturally aged mouse model to study age-related changes. Previous research has linked the age-related loss of bone trabeculae and the acceleration of osteoporosis development to increased lipid peroxidation and oxidized phospholipid synthesis (Palmieri et al., 2021). Our findings revealed that vertebral osteoporosis and disc degeneration are more severe in naturally aged mice than in young mice, supporting the notion that age and IDD are strongly associated (Teraguchi et al., 2017). Notably, MA administration improved both IDD and vertebral osteoporosis in aged mice.

While the primary functional component of the intervertebral disc is the NP, prior research on IDD has focused mostly on NP tissue changes (Sun et al., 2022), with less attention given to alterations in the AF and vertebral endplates. Recent studies suggest that the degradation of cartilaginous endplates is a key factor in the onset of IDD (Rade et al., 2018). Cartilaginous endplates, which are avascular structures, function as cushions for mechanical loads and conduits for nutrient exchange (Ge et al., 2021). Our findings align with studies showing that substances such as astaxanthin and pinocembrin protect vertebral cartilage endplates from oxidative stress and degeneration by activating the Nrf-2/HO-1 pathway, thereby delaying IDD progression (Wang et al., 2023; Yang et al., 2023). The degradation of cartilage endplates disrupts the nutrient supply to the IVD, exacerbating disc degeneration (Yuan et al., 2015; Määttä et al., 2014).

Previous studies in ovariectomized rats have demonstrated a strong association between IDD and bone loss (Xiao et al., 2018). Micro-CT analysis of the L4‒L5 segment in our natural aged model revealed significant changes in vertebral bone quality and structure. Aged mice presented localized bone fragmentation, sparse trabeculae, and variable endplate calcification. MA treatment increased the number of closed pores in the endplates, reduced the open porosity and total pore volume, and decreased osteoclast activity, as evidenced by TRAP staining. These results suggest that MA can impede cartilage endplate remodeling, reduce bone turnover, and enhance subchondral bone microstructure. Furthermore, these structural improvements in the bone and endplates help maintain the mechanical stability of the spine and delay the progression of IDD (Bellido et al., 2010). Moreover, this study also confirmed that MA could significantly inhibit the differentiation of RAW264.7 cells into osteoclasts through cellular experiments, and this study combined *in vitro* and *in vivo* experiments to confirm that MA could improve osteoporosis very well.

During the process of IDD, epigenetic changes occur within cells, such as DNA methylation, histone modifications, non-coding RNAs and so on (Li et al., 2022). These epigenetic changes will lead to alterations in cell functions, and consequently have an impact on the synthesis of the ECM within the IVD. The ECM is critical for maintaining intervertebral disc structure and function. Degeneration of gelatinous NP cells is a major factor in IDD and is characterized by changes in ECM composition and organization (Mouw et al., 2014). GO functional enrichment analysis revealed a correlation with processes such as ECM degradation and collagen catabolism. Immunohistochemical staining revealed greater expression of Collagen II and Aggrecan in the MA-treated group than in the senescent group, suggesting that MA helps preserve ECM integrity. *In vitro*, MA upregulated Aggrecan and Collagen II and downregulated MMP-3, MMP-9, p21 and p16 expression in senescent endplate chondrocytes, suggesting a protective effect on these cells.

As an essential part of the spine, the IVD serves to support the body while accounting for total stability and mobility. The ECM of the IVD is essential for preserving both its structure and functionality. To provide mechanical support, modify cellular behavior, and govern the biochemical and biomechanical features of the IVD, a complex network of macromolecules known as the ECM is assembled, comprising collagens, proteoglycans, glycoproteins, and other noncollagenous proteins (Zhou et al., 2023). IDD is characterized by key aspects such as changes in and disarray of the IVD matrix. Collagen II and proteoglycans, the primary constituents of the NP gel, are produced by ECM cells (Wu et al., 2018). The results of the GO functional enrichment analysis in this study suggest a correlation with biometabolic processes such as ECM degradation and collagen catabolism. Degeneration of the gelatinous NP is thought to be a major factor in IVD degeneration because it is necessary for disc function (Wang et al., 2019). The expression of Collagen II and Aggrecan was considerably greater in the MA-treated group than in the senescent group, according to immunohistochemical staining. The structural makeup of the NP in the senescent group changed dramatically, as shown by HE and safranin-O green staining. There were many fewer NP cells in the IVD, and some of the NP cells were replaced with cells that resembled chondrocytes and were arranged in clusters. The NP stroma around the cells had varying degrees of disorganized arrangement, broken fibrous rings, uneven distribution, and mucous-like degeneration, all of which were improved in the MA group. The discs of the senescent group presented a considerable reduction in collagen fibers and a decrease in endplate thickness, both of which were somewhat improved following MA therapy, according to the Masson staining data. *In vitro* RT-qPCR studies revealed that MA upregulated the expression of Aggrecan and Collagen II and downregulated the expression of MMP-3, MMP-9, p21 and p16 in senescent endplate chondrocytes, and SA‒β‒gal staining revealed that MA significantly reduced senescence in endplate chondrocytes, suggesting that MA may have a protective effect on senescent endplate chondrocytes.

**FIGURE 9:**
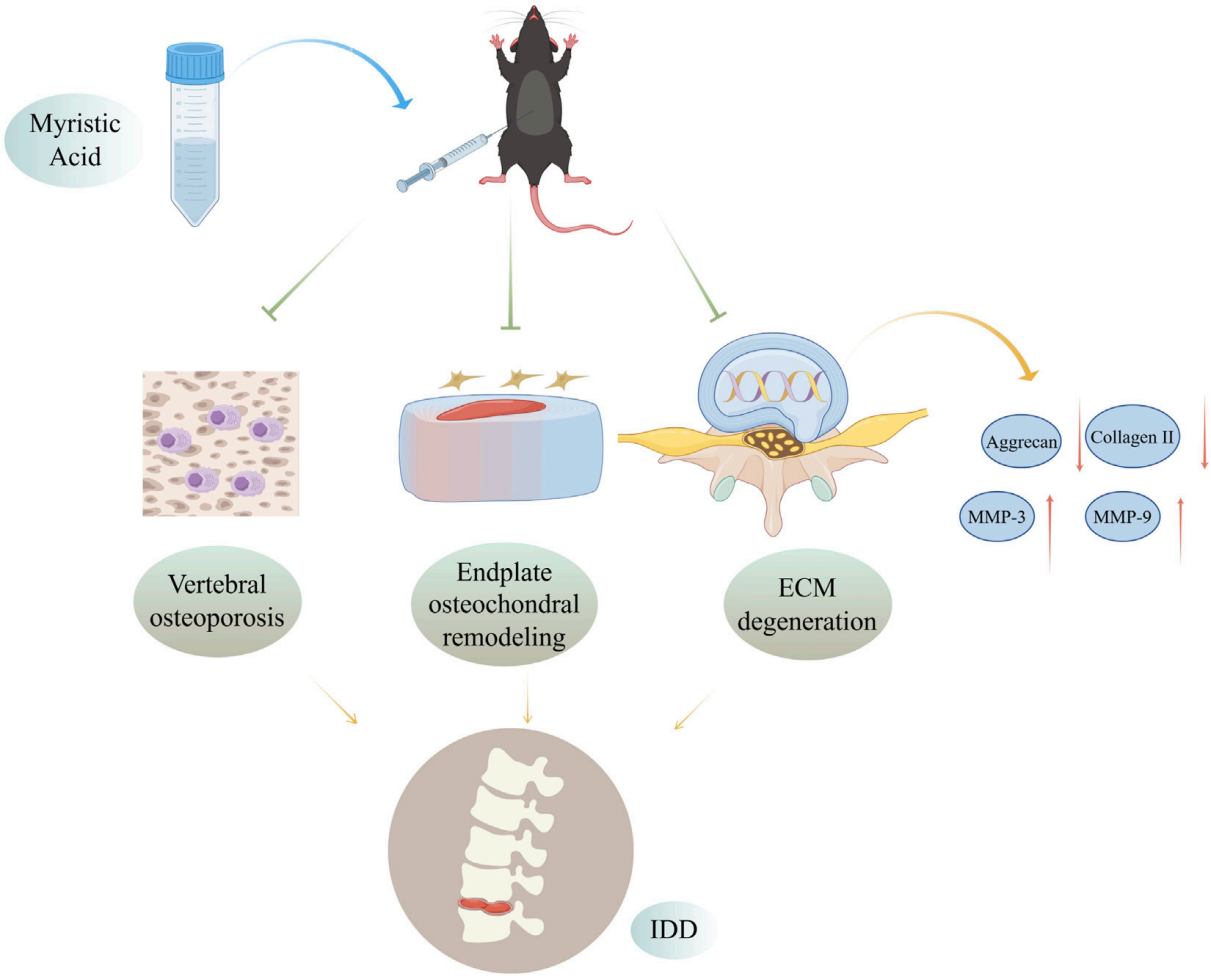
Mechanisms by which MA delays IDD by inhibiting endplate osteochondral remodeling and suppressing vertebral osteoporosis. MA inhibits vertebral osteoporosis, inhibits endplate osteochondral remodeling, and reduces extracellular matrix degradation in the intervertebral disc in naturally aged mice, thereby delaying disc degeneration. Figure was completed with the assistance of Figdraw (ID: ITPWSdda13).

Given that many traditional Chinese herbs and their extracts have shown strong therapeutic effects, there is increasing evidence supporting their use as potential treatment agents for IDD (Zhu et al., 2020). Our research revealed that the herbal monomer MA can delay disc degeneration by preventing vertebral osteoporosis and cartilage endplate remodeling (Figure 9). Our observations do, however, have certain limitations. For example, while we demonstrated that MA is not pharmacotoxic to the liver, heart, spleen, lungs, or kidneys of senescent mice, further research is needed to understand the long-term effects of MA. Additionally, the precise molecular mechanisms underlying the relationship between osteoporosis and IDD remain unclear and warrant further investigation. Future animal and clinical trials are necessary to confirm these findings and further explore the processes underlying the relationship between osteoporosis and IDD. Addressing osteoporosis and enhancing the structure of the vertebral body and endplates may help postpone IDD, but more research is needed to fully understand these mechanisms.

In conclusion, our study provides the first clear explanation of how MA prevents disc degeneration in mice naturally at this stage. The preventive impact of MA primarily prevents vertebral osteoporosis and endplate cartilage remodeling, preserving structural and functional integrity. These results might serve as a foundation for novel IDD treatment approaches. However, our findings are still preliminary. To assess the precise mechanism of action of MA in a dose-dependent and time-dependent manner more thoroughly, future research should concentrate on the dose of action, timing of MA administration, and signaling pathways involved in the relevant effects of MA. Additionally, the design of controlled trials should be improved.

## Data Availability

The data presented in this study have been deposited in the Dryad public database (Link: 10.5061/dryad.vhhmgqp4x).

## Glossary

MA: Myristic acid
RT-qPCR: Reverse Transcription Quantitative Polymerase Chain Reaction
qPCR: Quantitative Polymerase Chain Reaction
IDD: Intervertebral disc degeneration
LBP: Low back pain
NP: Nucleus pulposus
AF: Annulus fibrosus
ECM: Extracellular matrix
CEP: Cartilage endplate
EP: Endplates
IVD: Intervertebral disc
DHI: Disc height index
CEV: Cranial endplate volume
MMP-3: Matrix Metalloproteinase-3
MMP-9: Matrix Metalloproteinase-9
TRAP: Tartrate-Resistant Acid Phosphatase
TBHP: Tert-butyl hydroperoxide
H&E: Hematoxylin‒eosin
RANKL: Receptor Activator of Nuclear Factor Kappa-B Ligand
ACAN: Aggrecan
COL2A1: Collagen II
CCK-8: Cell Counting Kit-8
PBS: Phosphate-buffered saline
FBS: Fetal bovine serum
EDTA: Ethylenediaminetetraacetic acid
BV/TV: Bone volume/Total volume
(Tb.Th): Trabecular thickness
(Tb.N): Trabecular number
(Tb.Sp): Trabecular separation
(Po.N(cl)): Number of closed pores
(Po.V(tot)): Total volume of pore space
(Po(tot)%): total porosity percentage
