# Supplementary material for: Myristic acid beneficially modulates intervertebral disc degeneration by preventing endplate osteochondral remodeling and vertebral osteoporosis in naturally aged mice
Source: Front Pharmacol. 2025 Apr 1;16:1517221. doi: 10.3389/fphar.2025.1517221 (PMC12023261; doi:10.3389/fphar.2025.1517221)
Supplement: Supplementary file 1 [file Image1.pdf]

Supplementary Fig. 1

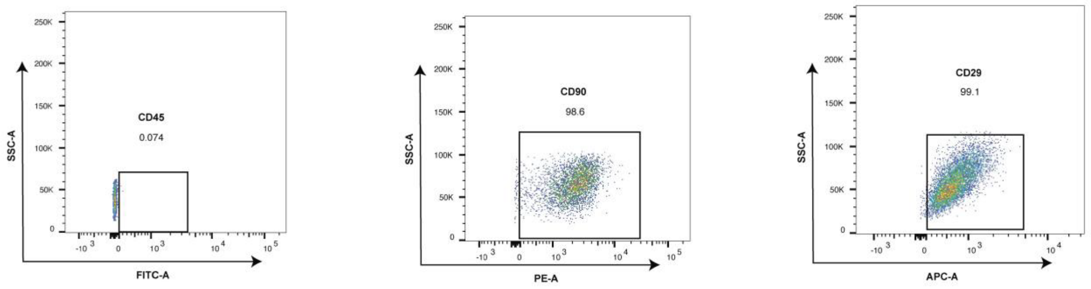

(Supplementary Fig. 1) We also performed flow cytometric characterization, and the results indicated that the screened endplate chondrocytes barely expressed CD45 and highly expressed CD29 and CD90, suggesting that the cells we extracted were EPCs.
